# Diagnostic value of cytokines in severe childhood *Mycoplasma pneumoniae* pneumonia combined with Adenovirus infection

**DOI:** 10.1186/s13052-024-01661-6

**Published:** 2024-05-07

**Authors:** Xiaowen Yi, Wanyu Jia, Wanying Li, Canyang Jia, Chunlan Song

**Affiliations:** Henan Province Engineering Research Center of Diagnosis and Treatment of Pediatric Infection and Critical Care, Children’s Hospital Affiliated to Zhengzhou University, Henan Children’s Hospital, Zhengzhou Children’s Hospital, Longhu Waihuan East Road, Zhengdong New District, Zhengzhou, Henan 450018 China

**Keywords:** Cytokines, *Mycoplasma pneumoniae*, *Adenovirus*, Mycoplasma pneumonia

## Abstract

**Background:**

To explore the alterations of inflammatory markers and immune-related cytokines in children infected with *Mycoplasma pneumoniae * (MP) combined with *Adenovirus* (ADV).

**Methods:**

The study population consisted of 201 children with MPP, and they were grouped according to whether they were coinfected with ADV infection and critically ill. Additionally, comparative analyses were performed. The diagnostic value of different indicators and combined indicators for SMPP combined with ADV was assessed using ROC curves.

**Results:**

There was no difference between group A1 and group A2, group B1 and group B2 in terms of age, gender, duration of hospitalisation and fever. The levels of calcitoninogen(PCT), lactate dehydrogenase concentration(LDH), interleukin(IL)-6, IL-8, IL-10, IL-4, IL-12P70, and IFN-γ in group A were higher than group B. The severe group (A1, B1) was significantly higher than the mild group (A2, B2) in terms of D-dimer, CRP, PCT, LDH, IL-6, IL-8, IL-10, IL-17a and number of patients with pleural effusion, solid lung changes. Among the individual indexes of D-dimer, CRP, N%,LDH, and PCT, the AUC of the combined test was 0.977, which was higher than that of the individual indicators. Among IL-6, IL-8, IL-10, and IL-17a, the AUC of the combined assay was 0.802, which was higher than that of the individual indicators.

**Conclusion:**

MP combined with ADV infection was associated with increased expression levels of IL-6, IL-8, IL-10, IL-4, IL-12P70, IFN-γ, and LDH. IL-6, IL-8, IL-10, IL-17a, LDH, PCT, CRP, and D-dimer could be used as predictors of SMPP and the combined test can improve the diagnostic value.

## Background

Pneumonia is a global concern, and even with widespread vaccination and updated treatment regimens, the morbidity and mortality rates of patients with pneumonia admitted to ICU are still as high as 30%, and those requiring invasive mechanical ventilation are as high as 35–50% [1]. In recent years, *Mycoplasma pneumoniae* (MP) has been one of the most common pathogens causing pneumonia, which is characterised by interstitial lung disease and can damage other organs through localized respiratory infections [2]. Symptoms of infection include fever, cough, sore throat, headache, and rhinitis, and can even be combined with severe lung diseases, such as pulmonary solid changes, pulmonary atelectasis, lung abscess, respiratory distress syndrome, and bronchiectasis [3]. Approximately 25–51% of children with Severe Mycoplasma pneumoniae pneumonia(SMPP) have combined with extrapulmonary manifestations [4]. In clinical practice, mixed infections of Mycoplasma pneumonia (MPP) are not uncommon, with *Adenovirus* (ADV) predominating, and airway occlusion caused by mixed infections of the two is an important cause of chronic airway disease in children, which affects the quality of life [5]. With the major outbreak of MP infections in 2023 and the widespread use of macrolides in children, some of these children are not effectively treated even after receiving one week of treatment, so the number of children with SMPP is increasing every year [6]. Therefore, it is important to explore the predictors of the development of MPP severe disease combined with ADV infection into severe disease. In most cases, the etiological diagnosis and outcome estimation of community acquired pneumonia (CAP) in children are unresolved issues. Although clinical guidelines for MPP have been updated, there are limited effective indicators for predicting the development and prognosis of patients. In recent years, the application of cytokine assays in the clinic has received increasing attention, and cytokine assays are now used in the assessment of immunological levels in clinical patients, and in the diagnosis of the disease and monitoring of therapeutic efficacy, which is especially important for early warning of patients with clinically severe infections [7, 8]. In this paper, we focus on the clinical factors and changes in the expression levels of relevant inflammatory cytokines in children with MP combined with ADV infection, with the expectation that the clinically relevant factors of SMPP can be detected as early as possible and intervened in order to reduce or shorten the course of the disease, thus reducing the mortality rate of the children.

## Methods

Two hundred and one children with MPP admitted to a large tertiary children’s hospital in Henan Province between June 2023 and December 2023 were retrospectively studied.The inclusion criteria for patients were as follows: met the diagnostic and treatment guidelines for *MPP* in children (2023 edition) [9], and had severe pneumonia cases were in accordance with the diagnostic and treatment guidelines for community-acquired pneumonia in children (2019 edition) [5].The exclusion criteria for patients were as follows: (1) aged > 16 years; (2) discharged and readmitted within 28 days; (3) received antibiotic treatment within 72 h prior to the patient’s hospitalization; (4) had parenchymal or interstitial changes in the lungs due to other diseases, such as lung cancer, tuberculosis, or pulmonary embolism; (5) had autoimmune system diseases or immunocompromised patients, such as HIV(+), splenectomy, organ or bone marrow transplantation, or receiving hormone or immunosuppressive therapy; (6) had other sites of infection; (7) had incomplete clinical data that could not be analyzed.

The grouping criteria for the enrolled children were as follows: Group A: MP combined with ADV and no other respiratory pathogen infections were detected during the same period; Group B: MP not combined with ADV; Group A1: MP combined with ADV severe disease; Group A2: pneumonia, MP combined with ADV mild disease; Group B1: MP not combined with ADV severe disease; and Group B2: MP not combined with ADV mild disease. Compliance with any of the following criteria suggested severe disease [5]: (1) persistent high fever (above 39 °C) for ≥ 5 days or fever for ≥ 7 days, with no downward trend in peak temperature (2). Patients who experienced extrapulmonary complications but did not meet the criteria for critical illness (3). Patients with a pulse oxygen saturation ≤ 93% at rest (4). Imaging reveals one of the following conditions: ≥2/3 of a single lung lobe is involved, and there is a homogeneous high-density solid lesion or high-density solid lesions in 2 or more lobes (regardless of the area of involvement), which may be accompanied by moderate to large amounts of pleural effusion and may be accompanied by a limited manifestation of fine bronchitis; Diffuse or bilateral fine bronchitis in ≥ 4/5 lung lobes, which may be combined with bronchiectasis and mucus plug formation leading to atelectasis (5). Patients with progressive exacerbation of clinical symptoms were diagnosed, and imaging showed that the extent of the lesion progressed by more than 50% in 24–48 h (6). Patients with significantly elevated CRP, LDH, and D-dimer levels. Criteria for mild disease: Patients who did not meet the criteria for severe disease.

### Data collection

Clinical characteristics, inflammatory indices (calcitoninogen PCT, C-reactive protein (CRP), white blood cell (WBC) count, neutrophil percentage N%, lymphocyte percentage L%, blood sedimentation, D-dimer, lactate dehydrogenase (LDH) and cytokines (ILs IL-1β, IL-2, IL-4, IL-5, IL-6, IL-8, IL-10, IL-17a, IL-12P70, tumor necrosis factor TNF-α, interferon IFN-γ, and IFN-α) were compared. The demographic characteristics, clinical features, and laboratory and imaging findings of all the children were measured as baseline data within 24 h of admission. All the acute-phase samples were collected before treatment with glucocorticoids and azithromycin, and all the specimens were collected and processed according to the kit instructions; all the procedures were performed by professional laboratory physicians.

### Statistical methods

The data of this study were statistically analyzed using SPSS 25.0 statistical software. The count data are expressed as percentages, and the χ2 test was used for comparisons between groups. Normally distributed and variance chi-square test data are expressed as the mean ± standard deviation (X ± S), and a t test was used for comparison; normally distributed data are expressed as M(P25,P75), and a nonparametric test was used for comparison. The diagnostic and predictive values of different indicators for severe MPP were evaluated using receiver operating characteristic (ROC) curves. *p* < 0.05 was considered to indicate statistical significance.

## Results

Clinical data: as shown in Table 1, there was no difference between groups A and B in terms of the duration of fever, hospitalization and coughing time of the children (*P* > 0.05), whereas Table 2 suggests that: there was a difference between groups A1 and A2, and B1 and B2 in terms of the number of days of hospitalization and duration of fever (*P* < 0.05), which were significantly higher in groups A1 and B1; there was no significant difference between groups A and B, A1 and A2, and B1 and B2 in terms of the number of children’s age, gender and other demographic characteristics were not significantly different (*P* > 0.05); the number of patients with pleural effusion and solid lung changes was significantly higher in groups A1 and B1 than in groups A2 and B2; As shown in Tables 1 and 2 there were no significant differences in D-dimer, CRP, and ESR between Groups A and B (*P* > 0.05); however, D-dimer and CRP were significantly greater in Group A1 than in Group A2 (*P* < 0.05) and significantly greater in Group B1 than in Group B2 (*P* < 0.05); moreover, the PCT and LDH concentrations were greater in Group A than in Group B, higher in Group A1 than in Group A2, and higher in Group B1 than in Group B2 (*P* < 0.05); WBC, N% and L% were not significantly different with respect to Groups A and B, B1 and B2, while N% and L% were significantly greater in Group A1 than in Group A2 (*P* < 0.05).

**Table 1:** Comparison of the general data, clinical characteristics and inflammatory indices of children in groups A and B

Form		Group A	Group B	*P*
Age (months)		72.69 ± 34.60	81.88 ± 34.86	0.062
Gender (n/%)	Male	58.00 (28.86%)	59.00 (29.35%)	0.346
	Female	46.00 (22.89%)	38.00 (18.90%)	
Lung consolidation (n/%)		51.00 (25.37%)	52.00 (25.87%)	
Pleural effusion (n/%)		28.00 (13.93%)	25.00 (12.44%)	
Length of stay M (P25,P75)		6.00 (5.00,8.00)	6.00 (5.00,8.00)	0.648
Heating time M (P25,P75)		7.00 (4.00,11.00)	6.00 (4.00,8.50)	0.314
Coughtime M (P25,P75)		8.500 (5.00,13.00)	7.00 (4.00,10.00)	0.093
D-dimer (ng/L)M(P25,P75)		0.43 (0.34,0.55)	0.41 (0.31,0.52)	0.315
WBC (*109/L) M (P25,P75)		8.87 (6.06,12.03)	8.10 (6.06,10.93)	0.318
N% M (P25,P75)		65.20 (55.48,74.40)	63.90 (53.30,73.55)	0.660
L% M (P25,P75)		28.75 (20.63,38.05)	28.50 (20.55,39.45)	0.901
CRP (mg/ml) M (P25,P75)		8.86 (1.50,20.32)	10.37 (2.58,23.91)	0.711
LDH (U/L) M (P25,P75)		319.00 (258.00,382.08)	272.00 (246.30,333.50)	0.000
ESR (x̄S)		30.44 ± 16.12	34.24 ± 19.16	0.129
PCT (ng/ml) M (P25,P75)		0.23 (1.14,0.67)	0.12 (0.06,0.33)	0.001

**Table 2:** Comparison of general information, clinical characteristics and inflammatory indices between children in groups A1/A2 and B1/B2

Form		Group A1	Group A2	*P*	Group B1	Group B2	*P*
Age (months)		76.78 ± 35.49	69.05 ± 33.68	0.258	87.33 ± 32.16	76.96 ± 36.75	0.145
Gender (n/%)	Male	27.00 (12.43%)	31.00 (16.43%)	0.777	27.00 (13.43%)	32.00 (15.92%)	0.783
	Female	22.00 (10.95%)	24.00 (11.94%)		19.00 (9.45%)	19.00 (9.45%)	
Lung consolidation (n/%)		33.00 (16.42%)	19.00 (9.45%)		30.00 (14.93%)	22.00 (10.95%)	
Pleural effusion (n/%)		16.00 (7.96%)	12.00 (5.97%)		20.00 (10.00%)	5.00 (2.49%)	
Length of stay M (P25,P75)		7.00 (6.00,10.00)	6.00 (4.00,7.00)	0.002	7.00 (5.00,9.00)	6.00 (5.00,7.00)	0.004
Heating time M (P25,P75)		7.00 (4.00,12.00)	6.00 (4.00,10.00)	0.180	7.00 (5.75,10.00)	5.00 (3.00,7.00)	0.002
Cough time M (P25,P75)		8.00 (4.00,12.00)	9.00 (5.00,13.00)	0.355	7.00 (4.75,11.25)	7.00 (4.00,10.00)	0.598
D-dimer (ng/L)M(P25,P75)		0.50 (0.39,0.72)	0.37 (0.31,0.48)	0.000	0.48 (0.38,1.31)	0.35 (0.29,0.46)	0.000
WBC (*109/L) M(P25,P75)		9.02 (6.68,12.27)	8.79 (5.47,11.96)	0.746	7.72 (5.95,10.61)	8.23 (6.22,11.34)	0.751
N% M (P25,P75)		68.80 (58.20,78.60)	59.70 (50.60,72.50)	0.008	66.90 (50.65,73.35)	63.80 (53.40,73.80)	0.874
L% M (P25,P75)		25.20 (17.30,34.50)	33.60 (22.30,45.60)	0.008	25.85 (19.45,38.90)	29.65 (21.83,39.88)	0.623
CRP (mg/ml) M (P25,P75)		14.04 (6.98,22.06)	6.51 (0.78,19.06)	0.004	14.31 (3.54,32.77)	7.77 (0.55,18.75)	0.038
LDH(U/L) M (P25,P75)		349.00 (288.00,464.50)	298.00 (258.00,350.00)	0.002	401.50 (294.00,653.60)	269.00 (237.00,308.00)	0.043
ESR (x̄ρ±S)		34.65 ± 15.83	26.69 ± 15.58	0.011	38.72 ± 19.62	30.2 ± 17.99	0.028
PCT (ng/ml) M (P25,P75)		0.17 (1.06,1,59)	0.07 (0.05,0.14)	0.000	0.10 (0.06,0.20)	0.20 (0.07,0.42)	0.024

Tables 3 and 4 suggest that IL-6, IL-8 and IL-10 levels were significantly greater in Group A than in Group B, Group A1 was greater than Group A2, and Group B1 was greater than Group B2 (*P* < 0.05); additionally, the cytokines IL-4, IL-12P70, and IFN-γ were significantly greater in Group A than in Group B (*P* < 0.05); additionally, the IL-17a level was significantly greater than that in Group A2, and Group B1 was greater than that in Group B2 (*P* < 0.05).

**Table 3:** Comparison of cytokine expression levels in children in groups A and B

Form M (P25,P75)	Group A	Group B	*P*
TNF-α(pg/ml)	1.64 (0.60,3.02)	2.08 (0.55,3.59)	0.506
IL-8 (pg/ml)	13.57 (9.57,18.74)	9.57 (6.84,14.08)	0.000
IL-6 (pg/ml)	5.70 (3.05,17.79)	3.87 (2.06,10.75)	0.010
IL-5 (pg/ml)	0.58 (0.36,0.98)	0.47 (0.27,0.78)	0.063
IL-4 (pg/ml)	2.36 (0.81,4.02)	1.81 (0.39,2.86)	0.013
IL-2 (pg/ml)	4.70 (2.53,6.74)	3.83 (1.88,6.05)	0.120
IL-1β (pg/ml)	4.275 (2.72,5.82)	4.32 (2.69,5.95)	0.915
IL-17a (pg/ml)	4.20 (1.19,8.16)	5.93 (1.28,10.15)	0.195
IL-12P70 (pg/ml)	2.72 (1.12,3.86)	1.94 (0.96,3.20)	0.038
IL-10 (pg/ml)	15.42 (8.90,25.63)	6.97 (3.96,9.12)	0.000
IFN-γ (pg/ml)	2.36 (0.95,6.31)	1.49 (0.59,2.60)	0.002
IFN-α (pg/ml)	2.92 (1.80,8.35)	2.64 (1.55,7.03)	0.192

**Table 4:** Comparison of cytokine expression levels in children in groups A1/A2 and B1/B2

Form (M(P25,P75))	Group A1	Group A2	*P*	Group B1	Group B2	*P*
TNF-α (pg/ml)	1.88 (0.66,2.86)	1.56 (0.48,3.12)	0.682	2.35 (0.86,3.72)	1.68 (0.53,2.94)	0.225
IL-8 (pg/ml)	14.92 (12.24,19.47)	11.67 (8.00,16.39)	0.001	11.70 (7.98,22.05)	8.91 (5.06,12.75)	0.004
IL-6 (pg/ml)	9.09 (3.34,25.99)	4.94 (2.64,11.44)	0.068	5.01 (2.54,22.82)	3.18 (1.70,6.99)	0.009
IL-5 (pg/ml)	0.58 (0.35,1.00)	0.56 (0.38,0.98)	0.964	0.42 (0.26,0.79)	0.54 (0.32,0.77)	0.215
IL-4 (pg/ml)	2.37 (1.14,3.83)	2.26 (0.70,4.20)	0.990	2.08 (0.70,2.80)	1.58 (0.38,2.98)	0.414
IL-2 (pg/ml)	5.27 (2.71,6.68)	4.38 (2.42,6.79)	0.752	4.02 (1.79,6.55)	3.68 (1.93,5.77)	0.742
IL-1β (pg/ml)	3.87 (2.64,6.12)	4.52 (2.78,5.70)	0.660	4.51 (3.04,6.20)	4.06 (2.33,5.48)	0.278
IL-17a (pg/ml)	5.22 (3.15,9.97)	1.77 (0.25,7.23)	0.000	10.16 (6.91,10.25)	2.70 (0.21,5.80)	0.000
IL-12P70 (pg/ml)	2.41 (1.14,3.86)	2.91 (1.07,4.10)	0.876	1.75 (0.88,3.19)	2.36 (1.26,3.28)	0.364
IL-10 (pg/ml)	23.08 (13.73,38.42)	11.84 (6.02,15.60)	0.000	8.29 (5.78,11.15)	6.02 (3.04,8.45)	0.001
IFN-γ(pg/ml)	2.60 (1.23,9.14)	2.22 (0.85,3.90)	0.244	1.72 (0.88,4.46)	1.28 (0.41,2.16)	0.091
IFN-α (pg/ml)	2.69 (1.85,6.04)	4.15 (1.77,11.97)	1.000	2.45 (1.50,6.04)	2.92 (1.69,7.26)	0.068

**Fig. 1:**
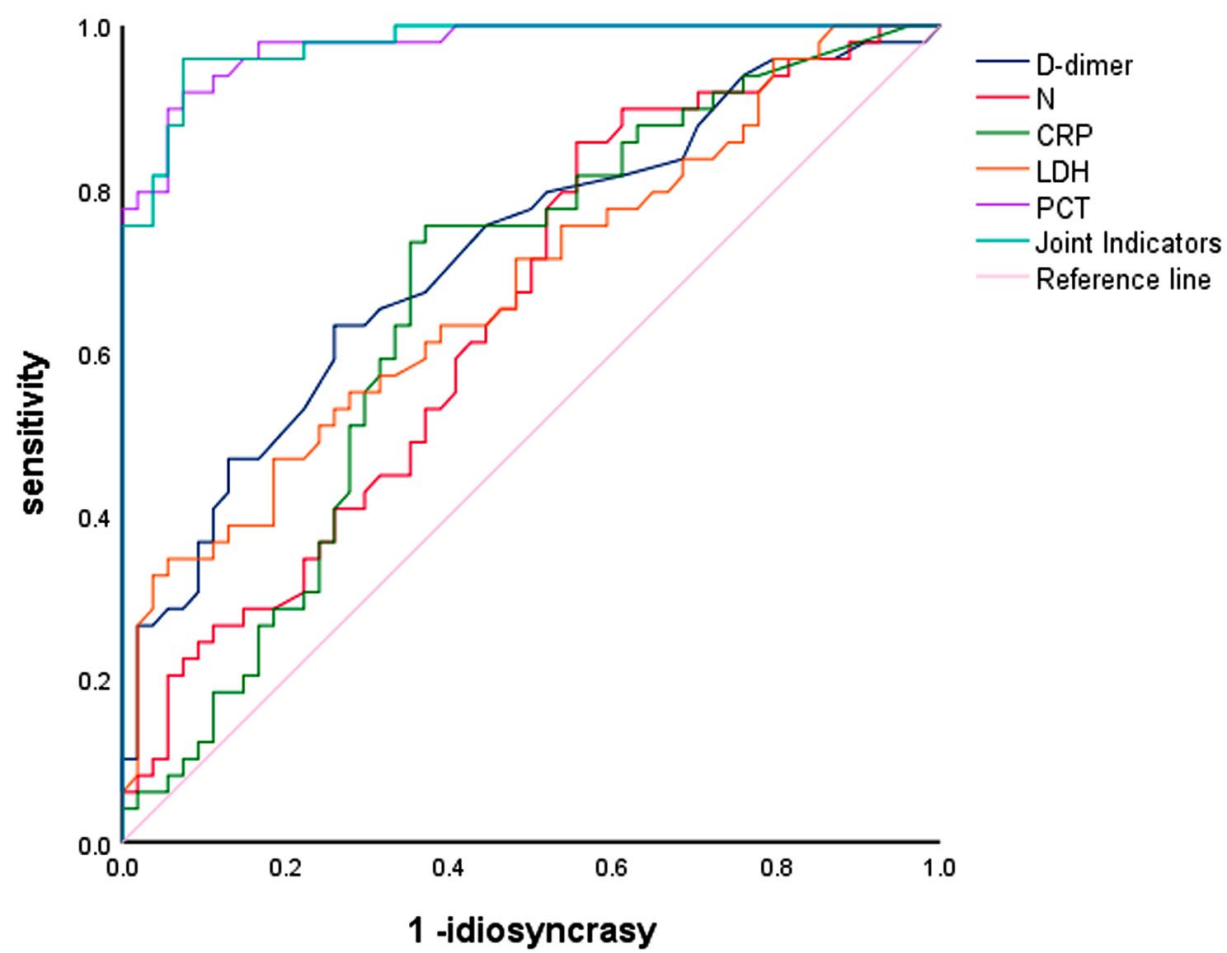
ROC curves for inflammatory indicators and combined indicators for predicting severe MPP

**Fig. 2:**
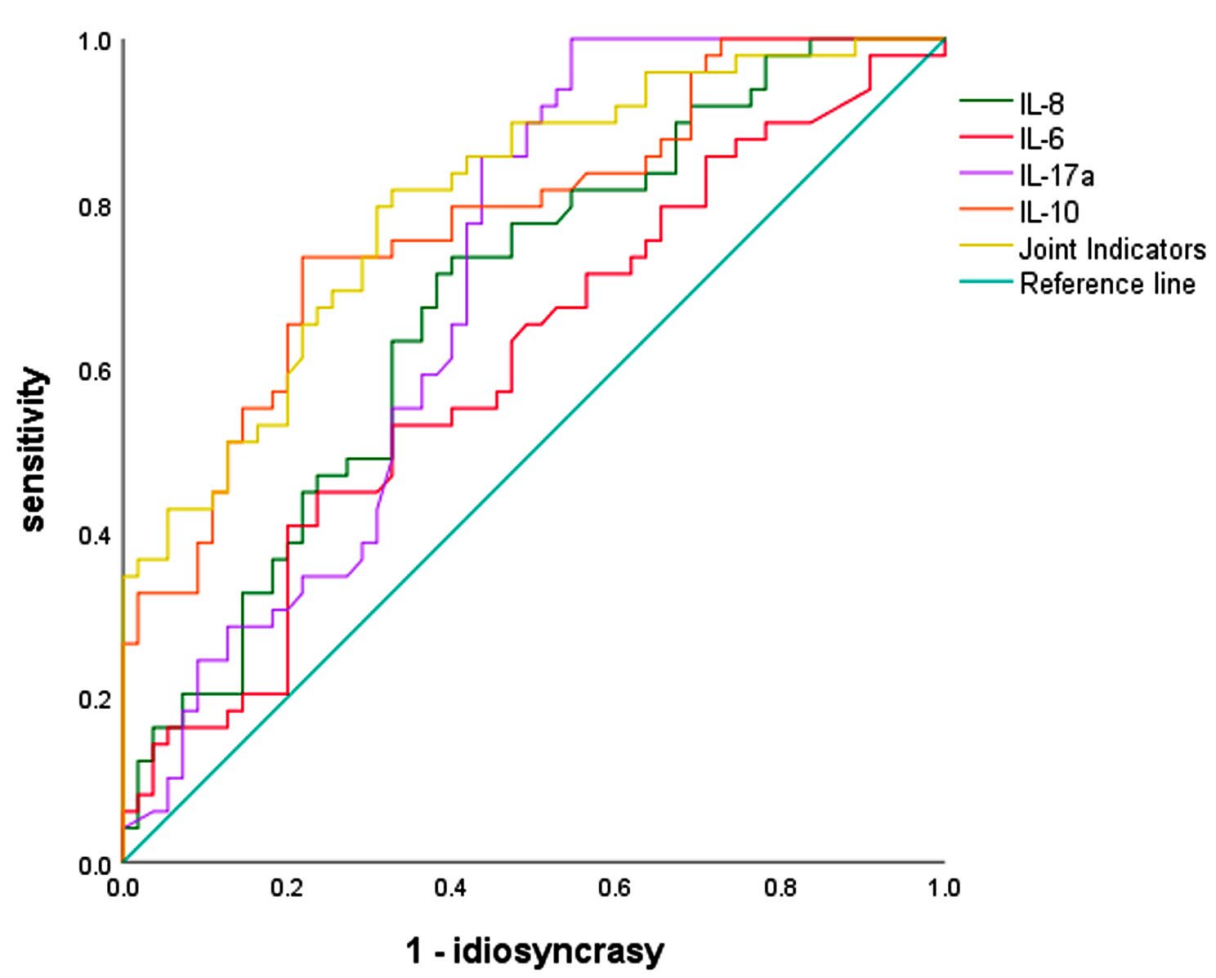
ROC curves of cytokines and combined metrics for predicting severe MPP

ROC curve analysis of the comparisons between groups A1 and A2 revealed that among the individual indices D-dimer, CRP, N%, LDH and PCT, the area under the curve (AUC) of D-dimer was 0.721, the AUC of CRP was 0.663, the AUC of N% was 0.647, the AUC of LDH was 0.68, and the AUC of PCT was the largest, 0.976, with 95% CI (0.952). CI of (0.952 0.999) is shown in Fig. 1, and Table 5 suggests that the AUC of 0.977 is greater than that of the single indicators when the above five indicators are tested in combination, which is more significant for the diagnosis of severe disease. The AUC of IL IL-6 was 0.604, the AUC of IL-8 was 0.681, the AUC of IL-17a was 0.704, and the AUC of IL-10 was the largest, 0.778, with a 95% CI of 0.69, 0.867, as shown in Fig. 2; Table 5. These findings suggest that when the above four indicators were tested in combination, the AUC was 0.802, which was greater than that of a single indicator and more significant for the diagnosis of severe disease. diagnosis of severe disease is more significant.

**Table 5:** Diagnostic significance of inflammatory indices, cytokines and combined indices in SMPP

	Cot-off	sensitivity	idiosyncrasy	Jordon index	AUC(95%CI)	*P*
D-dimer (ng/l)	0.47	0.633	0.741	0.374	0.721 (0.622,0.819)	<0.001
N (%)	57.15	0.857	0.444	0.301	0.647 (0.541,0.753)	0.01
CRP (mg/ml)	8.37	0.755	0.63	0.385	0.663 (0.557,0.769)	0.004
LDH (U/L)	398	0.347	0.944	0.291	0.680 (0.577,0.784)	0.002
PCT (ng/ml)	0.33	0.918	0.926	0.844	0.976 (0.952,0.999)	<0.001
Joint Indicators 1		0.96	0.93	0.89	0.977 (0.955,0.999)	<0.001
IL-8	12.63	0.735	0.6	0.335	0.681 (0.580,0.783)	0.001
IL-6	11.67	0.449	0.764	0.213	0.604 (0.495,0.713)	0.068
IL-17a	1.12	1	0.455	0.455	0.704 (0.603,0.805)	<0.001
IL-10	16.02	0.735	0.782	0.517	0.778 (0.690,0.867)	<0.001
Joint Indicators 2		0.898	0.473	0.371	0.802 (0.719,0.885)	<0.001

## Discussion

In China, MP usually leads to the highest incidence of atypical pathogen pneumonia.ADV is a highly contagious pathogen, and the inflammatory response triggered by its pathogenesis can exacerbate the damage caused by the body’s infection and lead to further deterioration of the disease, which is a serious threat to children’s health [10]. MP is a pathogen that can be detected by directly competing with or scavenging other bacteria [11, 12]. Viral infections can damage the pulmonary epithelium and suppress the immune response, promoting bacterial growth and leading to secondary bacterial infections [13]. Several studies have shown [14–16]that the rate of positive ADV tests is significantly greater in hospitalized children and that the disease tends to be more severe when MP is co-infected with ADV. The severity of disease in patients with pneumonia depends on two aspects, i.e., immune resistance and tissue repair ability [17]. The common clinical symptoms in children with SMPP are pleural effusion, lung solidity, pulmonary atelectasis, respiratory distress, and even complications such as other organ injuries, infections, and hypoxic death.The present study showed that the number of days of hospitalization and duration of fever in the A1 and B1 groups were significantly greater than those in the A2 and B2 groups. Similarly, the duration of hospitalization was longer in severely ill patients, and the number of patients with pleural effusion or pulmonary solid lesions was also greater than that in the mild group. Additionally, there were significant differences in the immune response and whether severe disease developed in children with MPP with or without ADV infection, and although current clinical biomarkers are beneficial for the diagnosis of CAP, many indicators are still difficult to distinguish between bacterial and viral infections or between mild and severe infections.

With in-depth research on severe pneumonia in recent years, the use of serological indicators for this disease has improved continuously.CRP is an acute time-phase reactive protein, which rises sharply when the body is subjected to external stimuli, and the reaction is particularly acute during the acute onset of the disease [18].PCT, a serum procalcitonin prepeptide substance, has a significant increase in serum concentration during bacterial infection, and during viral infection, the body releases gamma- interferon, on the other hand, exhibits an inhibitory effect on the release of calcitoninogen [19].D-dimer is able to suggest abnormalities secondary to fibrinolytic activity, and the activation of the coagulation system in children with severe pneumonia can have a direct impact on the diffusion function of the respiratory membranes and on alveolar gas exchange function [20].In this study, the values of D-dimer, PCT, CRP, blood sedimentation, and LDH concentration in the severe patients were significantly higher than those in the mild group; in a related study [21], it was also clearly proposed that there was a direct relationship between the levels of calcitoninogen, CRP, D-dimer, and IL-6 concentration in the children with SMPP and the incidence of severe disease, which is in agreement with this study. In addition, the present study showed that WBC, N% and L% are not significant in suggesting viral infection, but N% and L% were significantly elevated in children with severe MP combined with ADV infection. PCT and LDH were significantly elevated in combination with ADV infection, suggesting that PCT and LDH can be used as indicators of an abnormal state in organisms when they are subjected to external stimuli.

Cytokines are mainly synthesized and secreted by intrinsic and adaptive immune cells in the body as a class of molecular peptides or glycoproteins with multiple active functions. IL-6, whose main role is to regulate and promote the immune response, is the cytokine that is initially expressed by the intrinsic nervous system in response to injury and can be markedly increased if the body is subjected to an external stimulus. The overexpression of IL-10 inhibits the clearance of pathogens, thus leading to secondary bacterial lung infections [22]. IL-17a levels have been found to correlate with disease severity in related studies [23]. MIKACENIC et al. [24]showed that IL-17a levels were strongly associated with increased percentages of neutrophils and that elevated IL-17a levels correlated with increased SOFA scores. According to related studies [21], IL-6, IL-8, and IFN-γ are significantly elevated in ADV infection [12, 25], and IL-1α, IL-2, IL-4, IL-5, IL-6, IL-8, IL-17 A, IFN-γ, and TNF-α are significantly elevated in MP infection [12, 26–28]. Because MPP combined with ADV can exacerbate the condition of children with pneumonia, it is necessary to explore meaningful cytokines to improve diagnostic accuracy during this stage of research. The results of this study showed that the expression levels of IL-6, IL-8, IL-10, IL-4, IL-12P70, and IFN-γ were greater in patients with combined ADV infection, which have a certain diagnostic value. These results show that the immune system disorders occur in children with Mycoplasma pneumoniae pneumonia complicated with Adenovirus infection, which leads to increased cytokine expression levels, suggesting that the development of severe disease is more likely. Therefore, we suggest that more active treatment measures should be taken for these children in the early stage. Several studies have shown that the expression of cytokines in patients with pneumonia is pathogen specific and that the host immune system is activated by the cellular immune response during viral infection [29, 30]. This finding is consistent with the present study. The expression levels of IL-6, IL-8, IL-10, and IL-17a were significantly greater in patients with severe disease, which have a certain predictive effect on disease severity. Therefore, targeted therapy for the above cytokines has an important role in improving prognosis and treatment. Studies have shown [31] that preventing interleukin synthesis and antagonizing the biological activity of interleukin have a certain effect on the treatment of asthma and respiratory tract infectious diseases. Whether these drugs can be used in the treatment of severe pneumonia in the future needs further research.

In this study, ROC curve analysis of the comparisons between groups A1 and A2 showed that among the individual indicators of N%, LDH, PCT, CRP and D-dimer, the diagnostic value of PCT was greater than that of PCT when it was greater than 0.33 (ng/ml); the diagnostic significance of the simultaneous elevation of the five indicators was greater than that of the individual indicators; the diagnostic value of the elevated concentrations of IL-6, IL-8, IL-10 and IL-17a was greater; and the diagnostic value of the elevated concentrations of the four cytokines was greater than that of the individual indicators. IL-10 has greater diagnostic value than the other cytokines, and a concentration greater than 16.02 (pg/ml) suggests severe disease; elevated concentrations of these four cytokines have significantly greater significance in the diagnosis of severe disease than do the individual indicators. The ROC curve suggested that the combination of the above indicators could play a synergistic role in the diagnosis of severe MP pneumonia in children, and the diagnostic effect was ideal.

In addition, this study is a retrospective analysis with a small sample size, which has certain limitations, and the specific pathways affected by various cytokines during the course of MPP still need to be further explored. This study only studied the cytokine changes in Mycoplasma pneumoniae with Adenovirus infection, and we will also study the changes in other viral infections in the future.

## Conclusion

In conclusion, determining the changes in cytokine levels in children treated with MP combined with ADV is highly valuable. In this study, the serum inflammatory indices N%, LDH, PCT, CRP, D-dimer and the cytokines IL-6, IL-8, IL-10, and IL-17a could be used to predict severe disease, and the combined indices were of great value in the diagnosis of severe disease. In combined ADV infections, the expression levels of IL-6, IL-8, IL-10, IL-4, IL-12P70, IFN-γ and LDH were greater. In conclusion, this study provides an important reference for the immune status of children treated with MP-combined ADV, which will provide diagnostic significance for severe MP-combined ADV and can be used as a reference in clinical practice.

## Data Availability

The datasets used and/or analysed during the current study are available from the corresponding author on reasonable request.
